# Fearful arousals in sleep terrors and sleep-related hypermotor epileptic seizures may involve the salience network and the acute stress response of Cannon and Selye

**DOI:** 10.1016/j.ebr.2024.100650

**Published:** 2024-02-01

**Authors:** Péter Halász, Péter Simor, Anna Szűcs

**Affiliations:** a Szentágothai János Doctoral School, Semmelweis University, Budapest, Hungary; b Institute of Psychology, ELTE Faculty of Education and Psychology, Budapest, Hungary; c Institute of Behavioural Sciences Semmelweis University, Budapest, Hungary

**Keywords:** Sleep related hypermotor epilepsy, Sleep terror, Salience network, Sham alarm in sleep

## Abstract

We consider the disorders of arousal and sleep-related hypermotor epilepsy as genetic twin-conditions, one without, one with epilepsy. They share an augmented arousal-activity during NREM sleep with sleep-wake dissociations, culminating in sleep terrors and sleep-related hypermotor seizures with similar symptoms. The known mutations underlying the two spectra are different, but there are multifold population-genetic-, family- and even individual (the two conditions occurring in the same person) overlaps supporting common genetic roots. In the episodes of disorders of arousal, the anterior cingulate, anterior insular and pre-frontal cortices (shown to be involved in fear- and emotion processing) are activated within a sleeping brain. These regions overlap with the seizure-onset zones of successfully operated sleep-related hypermotor seizures, and notably, belong to the salience network being consistent with its hubs. The arousal-relatedness and the similar fearful confusion occurring in sleep terrors and hypermotor seizures, make them alike acute stress-responses emerging from sleep; triggered by false alarms. The activation of the anterior cingulate, prefrontal and insular regions in the episodes of both conditions, can easily mobilize the hypothalamo-pituitary-adrenal axis (preparing fight-flight responses in wakefulness); through its direct pathways to and from the salience network. This hypothesis has never been studied.

## Highlights

• DOA and SHE are parallel spectra; DOA without-, SHE with epilepsy.
• DOA and SHE share clinical symptoms, sleep/wake dissociation and brain localization.
• The shared brain-areas are the hubs of the salience network.
• Sleep terrors and hypermotor seizures may manifest sham-stress responses.

## Introduction: Sleep terror and sleep-related hypermotor epilepsy

**Fig. 1:**
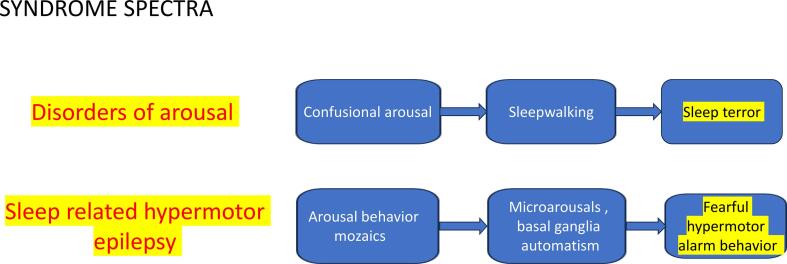
Parallel spectra of disorders of arousal and sleep-related hypermotor epilepsy. Their common event is a fearful arousal with sleep/wake dissociation from NREM sleep. The blue arrows signal he severity order of the episodes and the yellow highlights mark the most severe forms. (For interpretation of the references to colour in this figure legend, the reader is referred to the web version of this article.)

Fearful arousals arising from NREM sleep constitute two parallel conditions, one with- and one without epilepsy: sleep-related hypermotor epilepsy (SHE) and sleep terrors (ST) of the parasomnia group ‘disorders of arousal’ (DOA). (Fig. 1**)**.

Traditionally, confusional arousals, sleepwalking and sleep terrors are termed NREM parasomnias. Broughton [1] considered them as distinct entities, each representing a partial arousal from NREM sleep. This concept has changed to a spectral view of conditions related to disordered arousal [2]. Taking into account their similarity, the ICSD-3 included them in a single section as a continuum of disorders of arousal (DOA) [3].

DOA typically start in childhood with a prevalence ranging between 13 and 39 % that decreases with age to 1.6–4; and sleep terror in adulthood to 6.5 % [3], [4], [5], [6]. These conditions are more prevalent in children with epilepsy, attention-deficit hyperactivity disorder or developmental delays [7].

DOA, and in particular sleepwalking, are usually harmless in childhood but injurious or violent behaviors as well as frightening dreamlike experiences occur in the adult forms. Driving motor vehicles, attempts of suicide or even homicide raising forensic implications, have been described in sleepwalking adults [8], [9], [10], [11], [12], [13], [14], [15], [16]. The differentiation of DOA from SHE and REM sleep behavior disorder may be challenging Fig. 1.

Sleep deprivation is the most important trigger of DOA; provoking factors share a deepening and disorganizing effect on sleep. Fig. 1.

In sleep terrors, patients wake up screaming and disoriented, with dilated pupils, increased heart and respiration rate - the autonomic signs of fear; however, not remembering any dream contents. Anxiety and agitation may persist for 5–15 min after awakening. The events emerge from deep NREM sleep once/night, a more frequent night-occurrence is rare [1], [17], [18].

SHE may manifest in the form of minor motor events [13]; spells with motor automatisms (boxing, kicking or cycling indicating a fronto-striatal involvement) and a spectacular third type of seizures, where vehement alarm, panic-like behavior and important sympathetic activation occur [2]. These seizures with large chaotic or seemingly purposeful limb swings are alike fight-flight responses. Occasionally, patients would get out of bed and perform bizarre activities in a disoriented state manifesting the signs of fear unaware of its cause; and not remembering the seizures in most cases.

Most SHE cases are cryptogenic with electro-clinical syndromes alike the genetic forms [16]. In a minority of patients, there are mutations in the nicotinic acetylcholine receptor (NAChR) gene subunits and other mutations have been described as well [19], [20]. These patients make the group of autosomal dominant nocturnal frontal lobe epilepsy, which we consider as a model of arousal epilepsy [21].

The distribution of sleep related seizures carries the fingertips of sleep regulation. Seizures emerge from NREM sleep [17], [18]; two thirds of them appear from deep slow wave sleep during the first sleep cycle. The seizures associate with phasic slow wave activity mostly consistent with the cyclic alternating pattern A1-type [17].

Licchetta et al [22] studied the five years’ outcome in a large cohort of SHE patients. 86 % of them were sporadic cases and 16 % had underlying brain abnormalities. At the last assessment, 31 % of the patients achieved seizure freedom.

## Shared features of DOA and SHE

### Arousals during sleep

The events of both conditions link to arousals from slow wave sleep, strictly coupled with microstructural oscillations, such as the slow waves of the cyclic alternating pattern type A1. Both SHE and DOA episodes accumulate in the first and second sleep cycles when the homeostatic sleep pressure is the highest [23], [24]. The transit periods from the descending to the ascending slopes of the first sleep cycles (the latter preparing NREM to REM transitions) are critical in facilitating sleep dissociation, especially at the trough (the turning point from the descending to the ascending slope) of the cycle, where sleep promoting and arousal forces coexist (and “clash”) [25]. At this point, sleepers may change body position, and the high amplitude continuous slow wave oscillation disappears. These transitional periods, when sleep and arousal coincide, carry a risk of state dissociation, which might disinhibit emotional outbursts [26]. In one night, several seizures emerge, contrasting the rare DOA events. A somewhat dubious aspect is the age-distribution of the two spectra: DOA typically accumulate in childhood and manifest a descending age-slope to adulthood and older age [27]; this may be similar, but is certainly less clear in SHE [28], [29].

### Is cholinergic arousal the common root of DOA and SHE?

The convincing data about the shared arousal-relatedness and the similarities of DOA and SHE episodes and the gain-of function mutation of the NAChR gene subunit found in SHE patients suggest a common cholinergic origin of these conditions [24], [26].

### Genetic relations

There is a conspicuous familial accumulation of NREM parasomnias. Twin studies have shown a higher concordance rate for sleepwalking in monozygotic than dizygotic twins [30], [31], [32]. Based on the study of a four-generation family, Licis et al. [33] described the first genetic locus for sleepwalking at chromosome 20q12-q13.12 and suggested an autosomal dominant trait with reduced penetrance.

A shared genetic background of SHE and DOA has been evidenced by population-genetic data. A family study of 100 idiopathic frontal lobe epilepsy patients has revealed that 33 % of them had a history of parasomnias [16]. Another study [34] found that DOA was more frequent in idiopathic frontal lobe epilepsy patients' families, compared to the relatives of non-epileptic controls. Provini et al [15] found childhood parasomnias turning in the same persons, to idiopathic frontal lobe epilepsies later in life.

### Localization aspects: Sleep terrors‘ activation zones overlap with the seizure onset zones of sleep-related hypermotor seizures

SPECT, SEEG and current source mapping studies [35], [36], [37], [38] revealed a marked sleep state dissociation in sleep terrors: deep slow wave sleep in the fronto-dorsal regions paralleling an activated, wake-like state in the anterior cingulate, anterior upper insular and fronto-medial regions.

Retrospectively analyzing the seizure onset zones of successfully operated SHE cases between 1992 and 2020, we have found the overlap of seizure onset zones with the activated regions of sleep terror patients [39] Fig. 3.

**Fig. 2:**
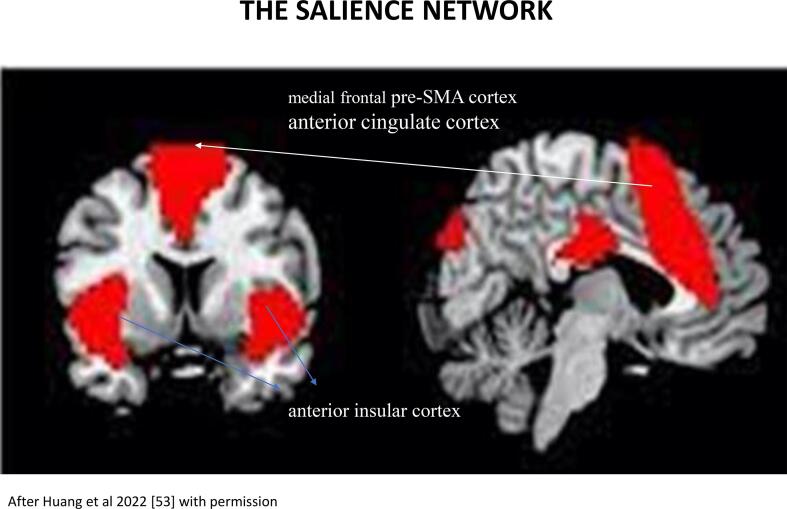
Representation of the salience network in bilateral dorsal anterior cingulate cortices, the anterior insula and the premotor medial frontal cortex anterior to the supplementary motor cortex.

**Fig. 3:**
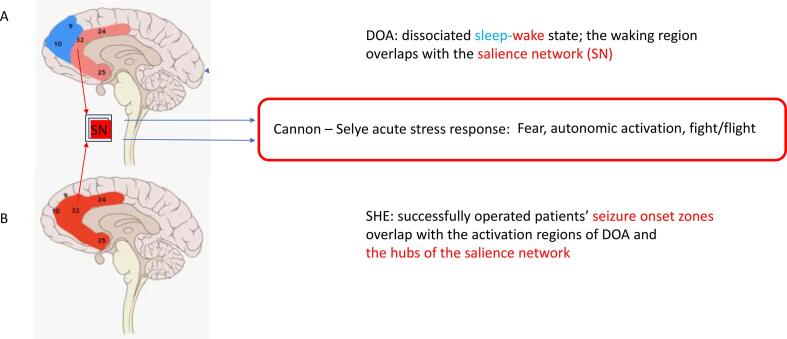
Overlap of the activation fields of night terrors, the seizure onset zones of hypermotor seizures and the salience network (SN) A) State dissociation between the sleeping fronto-dorsal region (blue) and the Activated anterior cingulate region (red). B) The successfully operated hypermotor seizures’ onset zones in sleep-related hypermotor epilepsy (red regions). Note the overlap between the A and B zones. The blue arrows mark that the activation of the salience network (SN) associated to the activation of the common key areas of sleep terrors and hypermotor seizures, may mobilize the Cannon-Selye acute stress response in sleep terrors and hypermotor seizures. (For interpretation of the references to colour in this figure legend, the reader is referred to the web version of this article.)

## Symptoms of hypermotor seizures, seizure onset zones, and classification trials

The symptoms and localization of hypermotor seizures explored by video EEG monitoring including stereo EEGs, is highly variable compared to the more stereotyped patterns of other epilepsies.

Rheims et al [40] described the semiology of hypermotor seizures (HMSs) in 31 patients. They made two groups. In group-1, negative emotional behaviors (fear, anger) and agitated but coordinated movements such as body rocking, pedaling, boxing, sitting or standing occurred. The authors note that”the mechanisms by which epileptic seizures may induce violent semi-purposeful agitation such as those observed in HMSs-1 remain unclear and mostly speculative. “ The seizure onset zones localized to the ventral prefrontal cortex. HMSs group-2 manifested tonic dystonic posturing with less agitation and emotions, and the seizure onset zones localized in the mesial premotor cortex. The mean duration of the hypermotor part of seizures was 22(3–53) sec, and it occurred at-, or within 10sec after seizure onset. Gibbs et al [41] studied the semiology of 135 hypermotor seizures in 91 frontal lobe-, and 44 extra-frontal SHE patients. They made four groups based on movement types. Type-1 involved elementary motor signs and asymmetric tonic posturing; type-2 had hypermotor features, axial tonic posturing with nonverbal vocalizations and facial contractions (pouting); type-3 presented with pedaling and stereotyped distal limb movements, speech, and manipulations; and type-4 presented with gestural and emotional signs of fear, and flight-fight behavior with autonomic symptoms. As a rule, the closer was the seizure onset zone to the primary motor cortex, the more elementary motor signs (contralateral jerks, tonic posturing and contralateral version) occurred; and the more anterior (close to the frontal pole, and orbitofrontal cortex) it was, the more complex movements and emotional signs manifested [40].

In seizures with coordinated behaviors and autonomic features, the discharges involved or interfered with the complex information processing network of the orbito-prefrontal, anterior cingulate, and temporal cortices [42]. Bartolomeil et al [43] reported a similar network behind ictal fear, anger and compulsive behaviors. Rylvin et al [44] have recognized a possible insular onset of HMSs with a frontal executive network. Ictal fear consistently involved the anterior ventral insula. In cingulate seizures, fear, vocalization, hypermotor phenomena, and pouting were typical [45]. Wan et al [46] provided an extensive review about SHE including localizations and sleep related ictal symptomatology as well as the commonalities between DOA and SHE.

## Discussion

### The overlap between the common activation regions of sleep terrors and hypermotor seizures in the prefrontal and cingulate negative emotion-processing areas as well as the salience network (SN)

It is remarkable, that the activation regions shared by sleep terror and hypermotor seizures - the anterior cingulate cortex, the anterior upper insular -, and the pre-supplementary mesial frontal cortex – are deeply involved in fear-, and emotion processing [47], [48], [49], [50] and overlap with the hubs of the salience network, normally signalizing meaningful events [51], [52], [53] Fig. 2.

### The participation of the emotion-processing prefrontal and insular regions, the salience network and the Cannon-Selye acute stress reaction in sleep terror and sleep-related hypermotor seizures

In 1932, Cannon coined the term „homeostasis” for the concerted physiological processes maintaining steady states in the living organism [54]. The „fight or flight” for animals’ behavioral and autonomic reaction to danger has been coined as an „acute stress response” in the „general adaptation syndrome - GAS”- of Selye [55].

The hypothalamo-pituitary-adrenal axis mobilizes the acute stress reaction including fight-flight behavior. The acute stress response involves several central nervous system regions; the endocrine-, hormonal and the autonomic nervous system.

A stressful event makes the hypothalamus stimulate the pituitary gland via neuro-hormones and the hormones synthetized in the anterior pituitary gland are conveyed to the adrenal cortex, producing mineralo-, and glucocorticoids, such as cortisol. Cortisol prepares the body for “fight or flight” and has other complex effects on several organ systems. The hypothalamus has neural connections to the autonomic nervous system including the adrenal medulla, producing catecholamine (epinephrine and norepinephrine) in response to stress [56], [58].

Based on the astonishing overlaps of the prefrontal and anterior cingulate regions known to process fear [57], the hubs of the salience network and the activation regions of both sleep terror and hypermotor seizures, we hypothesize that an inadequately mobilized acute stress reaction may be involved and shape the symptoms of both conditions. The escalation of cholinergic arousal to a fearful alarm behavior; as well as the intense autonomic and motor activation in both sleep terror and hypermotor seizures, may support this interpretation.

In hypermotor seizures, we assume the epileptic level over-activation of the sensitized arousal system. The anterior cingulate, prefrontal and insular seizure onset zones are overlapping - actually identical - with the hubs of the salience network and are involved in fear processing; linking to the hypopituitary-adrenal axis. Thus, the fear- and autonomic symptoms of hypermotor seizures may be consistent with an epileptic excitation that entrains a “secondary”, non-epileptic and false acute stress reaction, without any actual danger.

To our knowledge, this assumption - the salience network in direct connection with the HPA axis mobilizing the Cannon-Selye stress responses [54], [55] in both sleep related hypermotor seizures and sleep terrors - has never been studied.

Applying a system epilepsy approach, SHE can be interpreted as the epilepsy of the cholinergic arousal system built upon a gain of function mutation [16], [19], [26], [30].

### CRediT authorship contribution statement

**Péter Halász:** Writing – review & editing. **Péter Simor:** . **Anna Szűcs:** Writing – review & editing, Conceptualization.

## Declaration of competing interest

The authors declare that they have no known competing financial interests or personal relationships that could have appeared to influence the work reported in this paper.
